# Cholecystitis, Concealment of Hepatobiliary-Pancreatic Pathology: The Role of Endoscopic Retrograde Cholangiopancreatography and Endoscopic Ultrasound

**DOI:** 10.7759/cureus.20730

**Published:** 2021-12-27

**Authors:** Mansoor Zafar, Frederic Cuison, Oliver Shotton, Sara Lee, Samuel Vaughan, Tila Muhammad, Steven Fong

**Affiliations:** 1 Gastroenterology and Hepatology, Conquest Hospital, East Sussex Healthcare NHS Trust, St. Leonards-on-Sea, GBR; 2 Gastroenterology, Conquest Hospital, East Sussex Healthcare NHS Trust, St. Leonards-on-Sea, GBR; 3 General Internal Medicine and Gastroenterology, Conquest Hospital, East Sussex Healthcare NHS Trust, St. Leonards-on-Sea, GBR; 4 General Internal Medicine, Conquest Hospital, East Sussex Healthcare NHS Trust, St. Leonards-on-Sea, GBR; 5 Radiology, Conquest Hospital, East Sussex Healthcare NHS Trust, St. Leonards-on-Sea, GBR; 6 Gastroenterology and General Internal Medicine, Conquest Hospital, East Sussex Healthcare NHS Trust, St. Leonards-on-Sea, GBR

**Keywords:** endoscopic retrograde cholangiopancreatography, esophago gastroduodenoscopy, fine needle aspiration, computed tomography (ct ), magnetic resonance cholangiopancreatography (mrcp), endoscopic ultrasound (eus)

## Abstract

Cholecystitis is an inflammation of the gallbladder with classic symptoms of right upper quadrant abdominal pain and fever. The most common precipitating factor is cholelithiasis; however, it sometimes appears in conjunction with other hepatobiliary-pancreatic pathology. Management is generally done with antibiotics and supportive care with or without cholecystectomy. The surgical management in practice is often limited by surgery time and patient suitability considering their likely overall outcome. We have outlined two cases with different etiologies presenting as cholecystitis. The aim was to further understand the benefits of multidisciplinary team meetings to optimize patient care and emphasize the roles of endoscopic ultrasonography and endoscopic retrograde cholangiopancreatography in hepatobiliary pathology.

## Introduction

Cholecystitis is an inflammation of the gallbladder, which is most often secondary to obstructive gallstones (calculous cholecystitis) or, less frequently, independent of stones (acalculous cholecystitis). It commonly presents with acute right upper quadrant abdominal pain and fever. Initial management consists of antibiotics and supportive care, often requiring inpatient management. Best practice involves surgical removal of the gallbladder (cholecystectomy); however, the patient’s suitability for surgery or treatment before inflammation has settled will impact when and whether the surgery is performed [1]. In those who are not definitively managed with surgery, cholecystitis can recur, becoming chronic cholecystitis. A review of cohort studies showed the incidence of screen-detected gallstone disease in European populations to be 0.60%-1.39% per year [2].

In this report, we described two patients who presented with cholecystitis. Both had concomitant management challenges that arose in the form of associated hepatobiliary-pancreatic pathologies revealed throughout their treatment.

## Case presentation

Case 1

A 56-year-old woman presented to the emergency department with left-sided upper abdominal pain and yellow sclera. She also reported progressive on and off loose stools with a pasty consistency for the past month. There was no history of weight loss. She had a history of hypertension and type 2 diabetes mellitus. Her medication list included perindopril, dapagliflozin, ezetimibe, metformin, and insulin (28 units of Degludec at night and two to four units of Aspart three times a day after meals).

**Figure 1:**
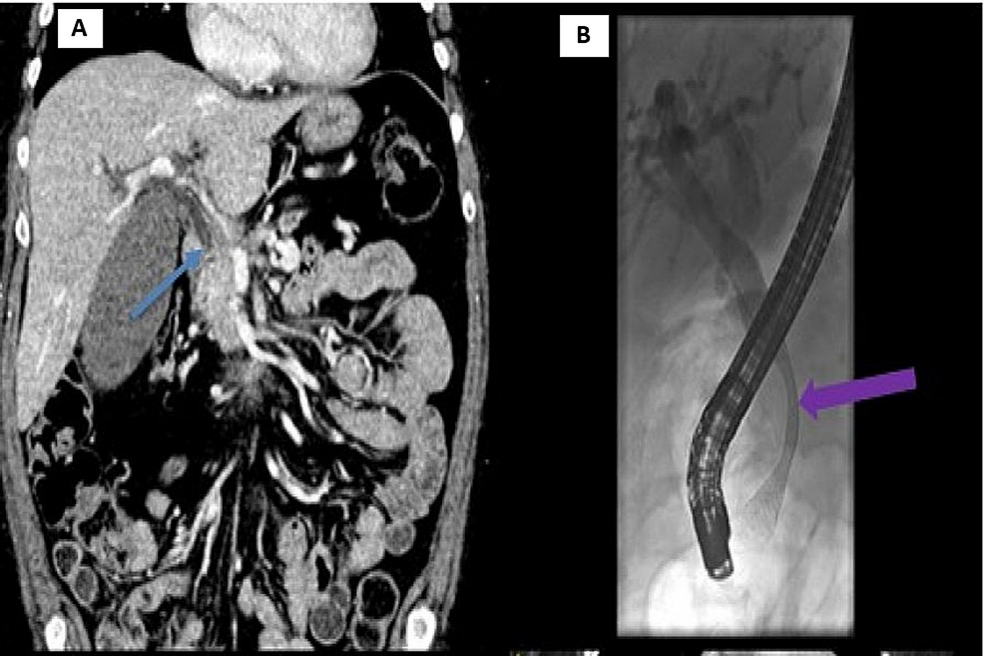
(A) Coronal CT of the abdomen with portal venous contrast, showing the initial presentation with dilated CBD and distinct transition (blue arrow), indicating obstruction secondary to a pancreatic head mass. (B) X-ray exposure from ERCP procedure. The patient was treated with an uncovered stent (purple arrow) deployed in the CBD via ERCP. CT, Computed tomography; CBD, common bile duct; ERCP, endoscopic retrograde cholangiopancreatography.

On examination, there were scleral icterus and mild tenderness along the epigastrium and left hypochondrium without signs of chronic liver disease or lymphadenopathy. Her initial blood results were total bilirubin of 121 µmol/L (0-21), alkaline phosphatase (ALP) of 651 U/L (30-130), alanine aminotransferase (ALT) of 920 U/L (10-35), and carbohydrate antigen (CA) 19-9 of 216 KU/L (0-34). Her stool cultures were negative. Fecal elastase was found to be low; she was started on Creon tablets. She subsequently had a computed tomography (CT) of the abdomen and pelvis with portal venous contrast, demonstrating a dilated biliary tree and distinct transition in the mid-common bile duct (CBD) secondary to a presumed compressive mass in the head and body of the pancreas (Figure 1). Her case was discussed in a multidisciplinary meeting (MDM), which concluded that she should be managed with biliary decompression via endoscopic retrograde cholangiopancreatography (ERCP). Cannulation occurred via the major duodenal papilla during this procedure, and an uncovered metal stent was inserted (Figure 1). Brushings were sent for histological analysis, the results of which were inconclusive. The patient was discharged with follow-up in an outpatient clinic with the hepatobiliary team to consider endoscopic ultrasound (EUS) for aiming biopsy or brushings.

**Figure 2:**
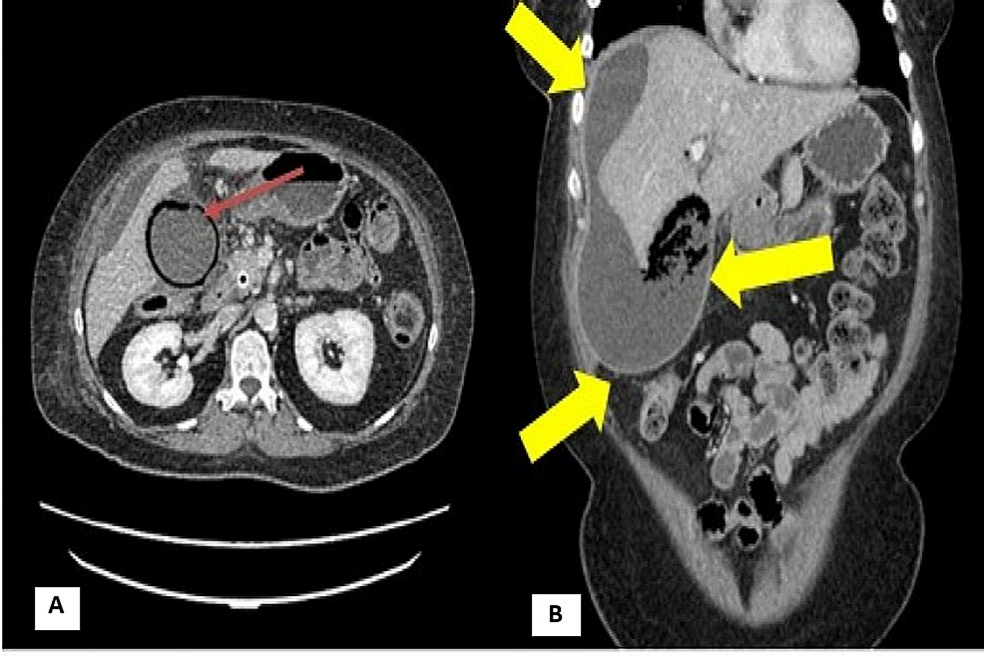
(A) Axial CT of the abdomen with portal venous contrast at the second presentation, showing emphysematous cholecystitis demonstrated by circumferential gas within the gallbladder wall (red arrow). (B) Coronal CT of the abdomen with portal venous contrast, showing the perihepatic collection (yellow arrows) secondary to gallbladder perforation as a complication of cholecystitis. CT, Computed tomography.

However, the patient presented sooner with recurrent pain in her upper abdomen three weeks later. An up-to-date CT of the abdomen and pelvis with portal venous contrast was performed, demonstrating locally perforated emphysematous cholecystitis continuous with a perihepatic abscess that extended superiorly to the sub-phrenic area (Figure 2).

**Figure 3:**
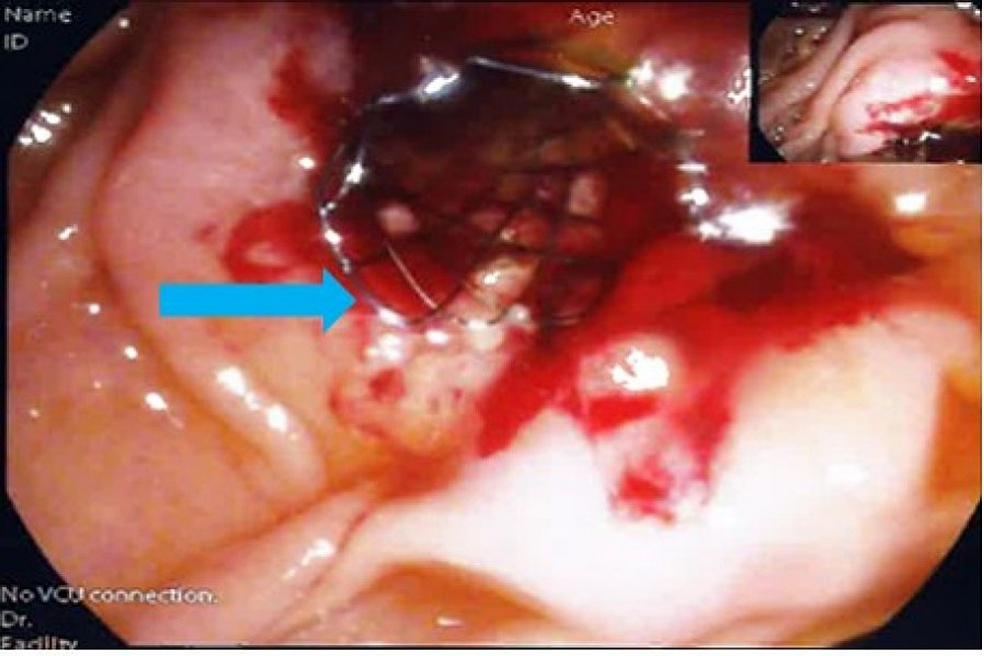
ERCP level 2 procedure Cannulation of the common bile duct was successfully performed via the major papilla. An uncovered metal stent (blue arrow) was placed in the common bile duct. ERCP, Endoscopic retrograde cholangiopancreatography.

She underwent EUS at a tertiary center, which revealed a mass in the head and neck of the pancreas around 3.7 cms in diameter. Fine needle aspiration (FNA) biopsies were sent for analysis during this procedure. The EUS also revealed proximal migration of the uncovered metal stent. The patient underwent a repeat ERCP (Figure 3) to replace this stent with both a newly uncovered metal and a plastic stent. Balloon trawls removed copious biliary sludge and pus during the procedure; however, the histological ERCP brushing taken was again inconclusive. The patient was managed with intravenous broad-spectrum antibiotics and radiologically inserted a percutaneous drain into the perihepatic collection. Following this, her liver function tests dramatically improved. The EUS-FNA biopsy characterized the mass as a mucin-producing neoplasm with mild focal atypia and no high-grade features. She was, therefore, discharged home with follow-up by the oncology team for consideration of ongoing chemotherapy.

Case 2

A 73-year-old woman presented to the emergency department with right upper quadrant abdominal pain. She had a past medical history of severe frailty, hypertension, uterovaginal prolapse, and restless leg syndrome. On examination, she had only mild tenderness in the right hypochondrium. A CT of the abdomen and pelvis with portal venous contrast was performed, demonstrating an enhancing thick-walled gallbladder containing luminal gas with pericholecystic inflammatory stranding fitting the clinical assessment of cholecystitis. The patient was managed on the surgical ward with broad-spectrum antibiotics and a radiologically inserted percutaneous cholecystostomy, from which a sample of pus was aspirated. Cultures of the drained fluid were positive for *Klebsiella*. After clinical improvement, she was discharged home following the removal of cholecystostomy drain, with a planned surgical outpatient follow-up.

**Figure 4:**
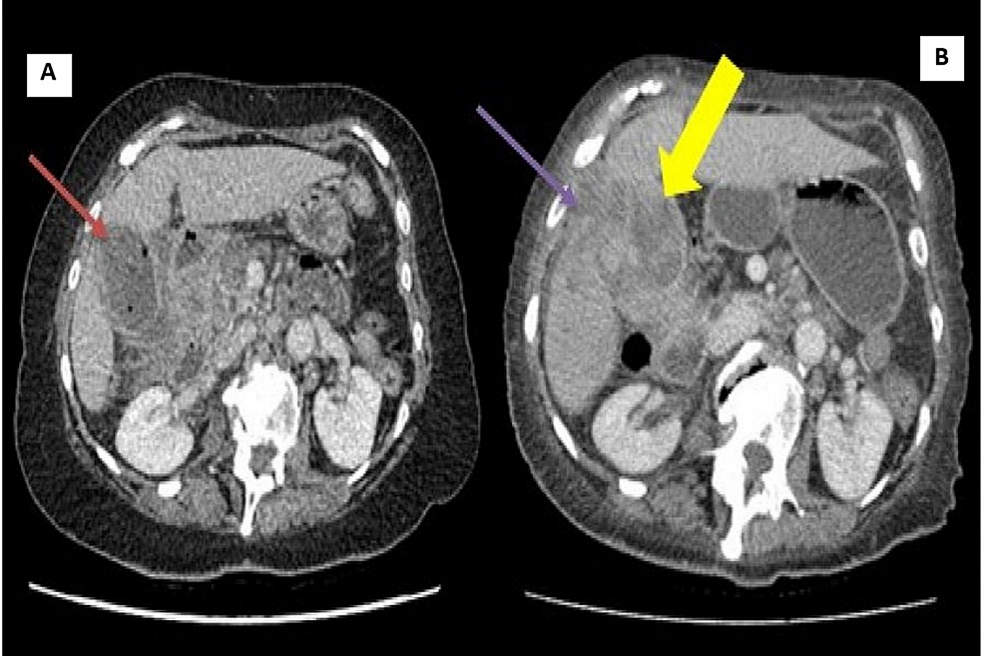
(A) CT of the abdomen with portal venous phase, showing the initial presentation with cholecystitis: thick-walled gallbladder (red arrow) containing gas locules with pericholecystic fat stranding and fluid. (B) CT of the abdomen with portal venous phase, showing the later presentation with extensive wall thickening of the gallbladder (yellow arrow) and inflammation into the adjacent liver (purple arrow). CT, Computed tomography.

Later, she had episodes of recurrent cholecystitis, and considering being a poor surgical candidate, these episodes were managed conservatively with intravenous fluid and antibiotics. However, six months later, she presented to the emergency department with recurrent pain in her right hypochondrium. Blood tests revealed hemoglobin (Hb) of 90 g/L (125-165), bilirubin of 28 μmol/L, ALP of 302 (30-130) U/L, and ALT of 18 (10-35) U/L. A CT of the abdomen and pelvis with portal venous phase was performed, which showed more extensive gallbladder wall thickening with some inflammation in the adjacent liver, suggestive of chronic cholecystitis (Figure 4).

**Figure 5:**
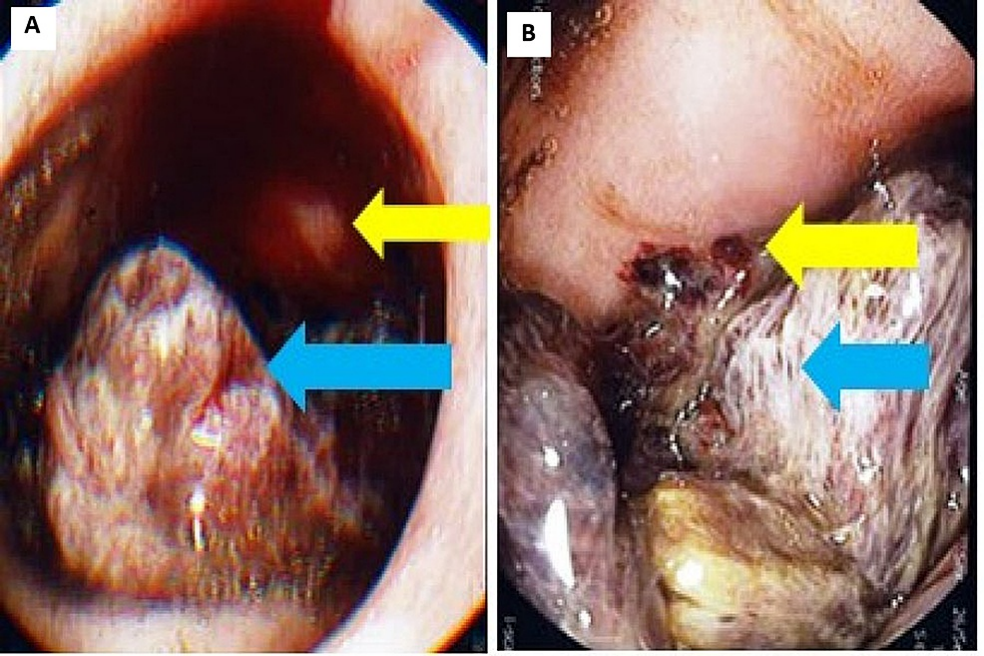
(A) A large irregular mass/tumor in the duodenal D1 area blocking views and entry into D2 (blue arrow). (B) The tumor is oozing blood (yellow arrow).

She was started on broad-spectrum intravenous antibiotics and intravenous fluid. However, during the third day of this admission, she had bleeding from upper gastrointestinal (GI) as coffee-ground vomiting and melena. Urgent blood tests showed Hb of 56 g/L (125-165) and required transfusion of four units of blood for stabilization. An urgent oral gastroduodenoscopy (OGD) was performed, revealing a bleeding mass in the duodenum, to which hemostasis was applied via clips and hemostatic spray (Figure 5).

**Figure 6:**
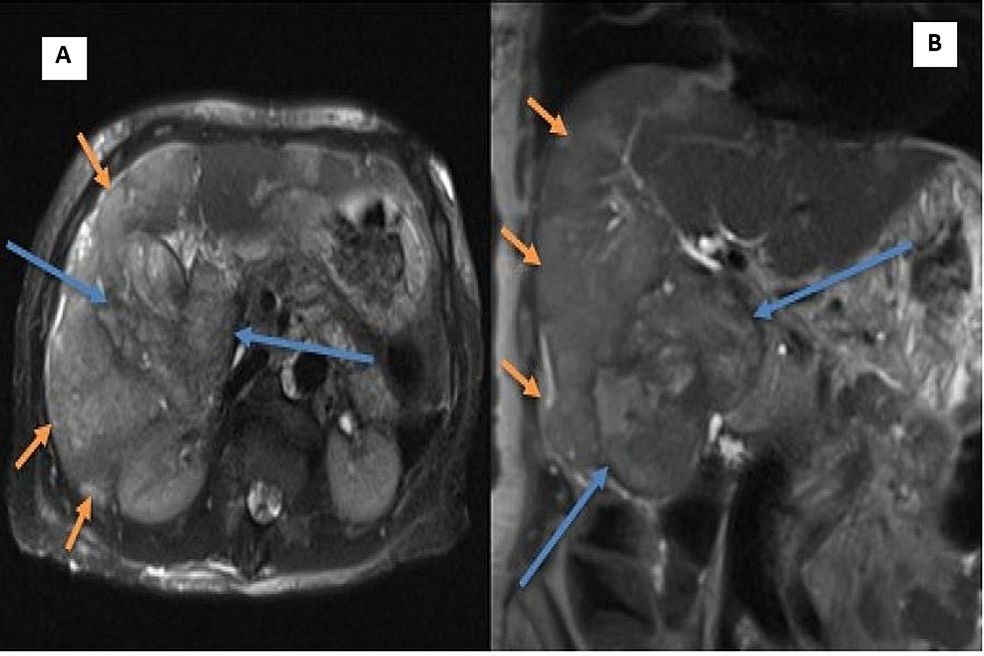
(A and B) T2 MRI images of the abdomen, axial and coronal, respectively, performed shortly after OGD, showing an extensive soft tissue within the gallbladder and its extension into the duodenum (blue arrows) and high signal within the liver from direct soft-tissue infiltration (orange arrows). MRI, Magnetic resonance imaging; OGD, oral gastroduodenoscopy.

Given the abnormal OGD and clinical findings, a magnetic resonance imaging (MRI) multiphase-contrast of the liver with MR-cholangiopancreatography (MRCP) was performed, demonstrating soft tissues throughout the gallbladder with local invasion to duodenum and liver, suggestive of malignancy (Figure 6). Her case was discussed in an MDM, which concluded that, given her severe frailty and likely metastatic cancer, she should be managed conservatively with the best supportive care. Therefore, she was palliated to hospice care and later, unfortunately, passed away.

## Discussion

Numerous studies have shown an association between the incidence of gallbladder disease and the development of other medical issues. For example, Kang et al. conducted a retrospective study on 93 patients, suggesting an increased incidence of gastric cancers in those with gallstones or cholecystectomies [3].

Furthermore, Nogueira et al. used the surveillance, epidemiology, and end results (SEER) Medicare-linked database (1992-2005), which included 17 cancer registries that cover approximately 26% of the US population, to identify that a history of gallstones and cholecystectomy were both associated with an increased risk of subsequent digestive tract cancers. These cancers included small intestinal carcinoids and non-cardiac gastric, liver, and pancreatic cancer. They also noted that colorectal cancer occurrence associated with gallstones or prior cholecystectomy decreased with increasing distance from the CBD. The results of this large US population-based study suggested that abnormalities in bile flow, such as bile reflux into the stomach, local inflammation (increased with gallstones and decreased with cholecystectomy), and changes in hormone levels, such as pancreatic cholecystokinin, are important in the etiology of digestive system cancer [4].

Thomsen et al. made a general population comparison of a cohort of 512,280 people collated using the Danish Civil Registration System [5]. They studied the incidence of cholecystitis in the two groups by linkage to the regional Hospital Discharge Registry. Their findings suggest that 230 diagnoses of cholecystitis were identified in the cancer cohort for 130,185 person-years (median follow-up time: 1.6 years), corresponding to an incidence rate of 1.8 per 1000 person-years. After adjustment for confounders, the relative risk (RR) for cholecystitis among cancer patients compared with the general population cohort was 1.38. Overall, the RR for cholecystitis was doubled during the first six months after a cancer diagnosis, after which the RR declined but remained greater than one throughout the rest of the follow-up period. Cancer patients between the ages of 51 and 70 years had the highest degree of increased risk of developing cholecystitis compared with other age groups. During the first six months after a cancer diagnosis, pancreatic cancers (12 cholecystitis events) and colorectal cancers (10 cholecystitis events) were associated with the greatest cholecystitis risk increase compared with other tumor types. After six months, most cancers were associated with a relatively small increased risk, although there was an RR of 4.72 among thyroid cancer patients (based on five cholecystitis events) [5].

Kalima et al. have suggested that bile reflux into the stomach is a common side effect of cholecystectomy [6]. Miwa et al. have shown that bile reflux has the potential to induce gastric adenocarcinomas in rats [7]. Pomare et al. have shown that the small intestine is also exposed to increased bile acids after cholecystectomy [8]. Studies have suggested that bile exposure modifies intestinal mucosal morphology and stimulates epithelial cell proliferation [9,10]. Additionally, inflammation plays a role in the association of gallstones with pancreatic cancer; hence, gallstones can lead to pancreatitis [11], which is a known risk factor for pancreatic cancer [12]. Shebl et al. have outlined an association between gall bladder stones with gall bladder cancer [13].

Studies have shown an association of cholecystitis with other gut and non-gut malignancies. We noticed that patients with symptoms and signs of cholecystitis were surrounded by the symptom complex associations with complex situations. This included pancreatitis as a sequela of a metastatic gall bladder malignancy that presented months earlier as cholecystitis was merely the tip of the iceberg. The use of MRCP imaging revealed a catastrophic outcome. We attempted to highlight the complexities that can arise when managing gallbladder diseases, such as cholecystitis, particularly acalculous cholecystitis, due to their association with numerous other diseases that may arise in conjunction with, before, or after their presentation. Lastly, EUS is important in the successful acquisition of biopsy samples and ERCP to perform stenting with decompression of CBD.

## Conclusions

These cases demonstrate how these associated diagnoses can alter the required management of cholecystitis and gallbladder disease and the importance of remaining observant of associated pathologies when managing patients presenting with symptoms of cholecystitis. MRCP is a more effective non-invasive modality to understand the pathology before invasive treatment. Although EUS is a better modality to obtain biopsy samples, the role of ERCP remains important to insert stents in CBD.
